# Polynuclear Silver(I)–Quinoxaline Complex: Comprehensive Structural Characterization, Antimycobacterial Properties and DNA/BSA Binding Study

**DOI:** 10.3390/pharmaceutics18020169

**Published:** 2026-01-27

**Authors:** Ghada Bouz, Nevena L. Stevanović, Marta Počkaj, Tina P. Andrejević, Iztok Turel, Ondřej Jand’ourek, Klára Konečná, Žiko Milanović, Kristina Milisavljević, Biljana Đ. Glišić

**Affiliations:** 1 Faculty of Pharmacy in Hradec Králové, Charles University, Akademika Heyrovského 1203, 500 03 Hradec Králové, Czech Republic; ghada.bouz@ffns.ac.rs (G.B.); jando6aa@faf.cuni.cz (O.J.); konecna@faf.cuni.cz (K.K.); 2 Faculty of Pharmacy, University of Business Academy in Novi Sad, Heroja Pinkija 4, 21000 Novi Sad, Serbia; 3 Department of Chemistry, Faculty of Science, University of Kragujevac, R. Domanovića 12, 34000 Kragujevac, Serbia; nevena.stevanovic@pmf.kg.ac.rs (N.L.S.); tina.andrejevic@pmf.kg.ac.rs (T.P.A.); 4 Faculty of Chemistry and Chemical Technology, University of Ljubljana, Večna pot 113, SI-1000 Ljubljana, Slovenia; marta.pockaj@fkkt.uni-lj.si (M.P.); iztok.turel@fkkt.uni-lj.si (I.T.); 5 Department of Science, Institute for Information Technologies Kragujevac, University of Kragujevac, Liceja Knezevine Srbije 1A, 34000 Kragujevac, Serbia; kristina.milisavljevic@pmf.kg.ac.rs

**Keywords:** *N*-substituted quinoxaline-2-carboxamides, silver(I) complexes, antimycobacterial activity, DNA interaction, protein interaction

## Abstract

**Background**: Silver(I) complexes with aromatic heterocyclic ligands are well known for their broad antimicrobial potential, largely attributed to their ability to interact with biomolecular targets. **Results and Discussion**: In this study, a new polynuclear silver(I) complex with *N*-(3′-phenylpropyl)quinoxaline-2-carboxamide (pqx-2ca), [Ag(NO_3_)(pqx-2ca)]_n_, was synthesized. Its structure was confirmed by single-crystal X-ray diffraction and comprehensively characterized using NMR, IR, and UV–Vis spectroscopy, while its behavior in solution was further elucidated through density functional theory (DFT) calculations combined with spectral simulations. The complex demonstrated significantly enhanced antimycobacterial activity compared with the free ligand when tested against the avirulent *Mycobacterium tuberculosis* H37Ra, fast-growing model organisms *M. smegmatis* and *M. aurum*, as well as the nontuberculous species *M. avium* and *M. kansasii*. Experimental and docking studies confirmed stable binding of the complex to subdomain III of bovine serum albumin (BSA) and to the minor groove of DNA. Furthermore, docking to validated mycobacterial targets revealed inhibitory potential toward the InhA and MmpL3 proteins, with binding affinities comparable to those of standard inhibitors. **Conclusions:** These results highlight [Ag(NO_3_)(pqx-2ca)]_n_ as a promising candidate for the development of silver-based antimycobacterial agents with a dual mechanism of action involving both DNA and protein targets.

## 1. Introduction

The medical applications of silver and its various compounds as antiseptic, antibacterial, and anti-inflammatory agents have been extensively documented throughout history, owing to the well-established antimicrobial properties of Ag(I) ions, which are closely associated with their ability to interact with protein thiol groups and nucleic acids [1,2,3]. This observation is consistent with the long-standing and clinically validated use of silver(I) sulfadiazine as one of the most effective topical therapeutic agents for the prevention and treatment of burn wound infections [4]. Numerous studies have further demonstrated that the pharmacological potential of many *N*-heterocyclic compounds can be substantially enhanced through coordination with Ag(I) ions. The antimicrobial activity of a silver(I) complex critically depends on the nature of the donor atoms bound to the metal center. It has been observed that silver(I) complexes containing weaker Ag–N and Ag–O bonds generally display a broader and more potent antimicrobial spectrum compared with those containing Ag–S and Ag–P bonds, the latter typically exhibiting markedly reduced or even negligible biological activity [5,6,7,8]. In this manner, rapid dissociation of Ag(I) ions from the complex is effectively prevented, thereby enabling a sustained and controlled release of the metal ions. This mechanism ensures prolonged and enhanced biological activity of the complex, as exemplified by silver(I) complexes with strongly coordinating *N*-heterocyclic carbene ligands (Ag-NHCs). Such controlled-release behavior not only improves the antimicrobial efficacy of these complexes but also has important implications for their potential therapeutic applications, including antimicrobial and anticancer strategies [9]. Beyond their established antimicrobial properties, Ag-NHC complexes have demonstrated significant in vitro cytotoxicity against multiple cancer cell lines, including ovarian (OVCAR-3) and breast (MB157) carcinoma cells, highlighting their potential as promising candidates for anticancer drug development [10].

**Figure 1:**
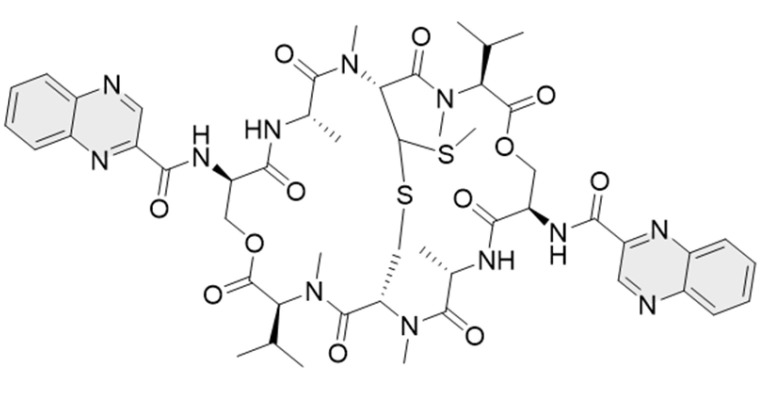
The chemical structure of echinomycin. Quinoxaline moiety is shown in gray.

Among the structurally diverse *N*-heterocycles investigated in coordination chemistry, quinoxaline and its derivatives occupy a particularly prominent position. This is largely attributed to the presence of two nitrogen atoms within the benzo-fused diazine ring, which enables their coordination to transition metal ions, as well as their intrinsic ability to interact with a wide range of biological targets [11]. Although naturally occurring quinoxaline derivatives are relatively scarce [12], their synthetic analogues have attracted considerable attention in recent years owing to their remarkably broad spectrum of biological and pharmacological activities. Numerous studies have demonstrated that quinoxaline-based molecules exhibit potent antimicrobial, antiviral, anticancer, anti-inflammatory, and antiparasitic properties, making this heterocyclic scaffold highly valuable for drug discovery and medicinal chemistry applications. A well-known representative is echinomycin, a naturally occurring quinoxaline antibiotic that exhibits strong activity against Gram-positive bacteria by intercalating into DNA and inhibiting transcription processes (Figure 1) [13]. It is worth noting that quinoxaline is a structural isomer of naphthyridine, differing in the arrangement of nitrogen atoms within their bicyclic ring structures. Both compound classes are important scaffolds in medicinal chemistry, as their core structures can bind to a wide range of biological targets and exhibit broad biological activity [14]. Beyond biomedical applications, one of the most important uses of transition metal complexes is in catalysis, where the coordinated ligand plays a crucial role in the design of catalysts for sustainable industrial processes [15,16]. In addition, transition metal complexes can be employed for the chemical modification of small molecules within the coordination sphere, which is impossible or significantly hindered in the absence of a metal center [17].

The structural versatility and ease of functionalization of the quinoxaline core further enable the rational design of novel derivatives with enhanced selectivity and reduced toxicity, which continues to stimulate intensive research efforts in this field. Furthermore, certain synthetic quinoxaline-2-carboxylic acid esters have demonstrated selective antimycobacterial activity, notably against *Mycobacterium tuberculosis* H37Ra, an effect attributed to the in situ reduction in the acetoxy group to the pharmacologically active free hydroxy form [18]. Considering the abovementioned factors, in the present study, we decided to use one synthetic quinoxaline derivative, *N*-(3’-phenylpropyl)quinoxaline-2-carboxamide (pqx-2ca) [19], for the synthesis of a new silver(I) complex, [Ag(NO_3_)(pqx-2ca)]_n_. We hypothesized that coordination of this ligand to the Ag(I) ion would yield a structurally well-defined silver(I) complex with significantly enhanced antimycobacterial activity compared with the free ligand. We further hypothesized that this activity is supported by a dual biomolecular targeting mechanism, involving stable interactions with DNA (preferentially via groove binding) and serum albumin, together with energetically favorable binding to key mycobacterial enzymes involved in mycolic acid metabolism, namely InhA and MmpL3. The synthesized complex was characterized using spectroscopic (^1^H and ^13^C NMR, IR and UV-Vis) and electrochemical (cyclic voltammetry) methods, while its solid-state structure was determined by single-crystal X-ray diffraction analysis. Furthermore, the pqx-2ca ligand and its silver(I) complex were evaluated for in vitro antimycobacterial activity against five mycobacterial strains: the avirulent *M. tuberculosis* H37Ra, the fast-growing model organisms *M. smegmatis* and *M. aurum*, as well as the nontuberculous *M. avium* and *M. kansasii*. The avirulent *M. tuberculosis* H37Ra closely mirrors pathogenic *M. tuberculosis* metabolically and structurally, making it a useful low-biosafety surrogate; however, its slow growth remains a limitation for routine use [20]. Fast-growing, nonpathogenic models (*M. smegmatis* and *M. aurum*) offer rapid, cost-effective alternative; nevertheless, their greater phylogenetic distance from *M. tuberculosis* may reduce predictive accuracy [21,22]. Nontuberculous mycobacteria (*M. avium* and *M. kansasii*) share intrinsic drug tolerance and envelope characteristics with *M. tuberculosis*, cause infection courses similar to that of *M. tuberculosis*, and are therefore valuable for assessing spectrum of activity [23]. Together, this five-mycobacterial panel enables efficient screening of antimycobacterial activity while maintaining biological relevance. The DNA/BSA binding properties of the compounds were investigated using fluorescence emission spectroscopy.

## 2. Results and Discussion

### 2.1. Synthesis of the Silver(I) Complex

**Scheme 1:**
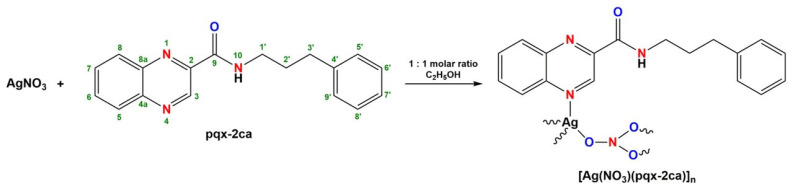
Schematic presentation of the reaction route for the synthesis of the silver(I) complex. Atom numbering in the ligand follows IUPAC recommendations and is used for NMR characterization of the synthesized complex.

A polynuclear silver(I) complex, [Ag(NO_3_)(pqx-2ca)]_n_, was synthesized by reacting AgNO_3_ with an equimolar amount of *N*-(3’-phenylpropyl)quinoxaline-2-carboxamide (pqx-2ca) in ethanol (96%) at room temperature in good yield (Scheme 1). The synthesized complex was characterized by elemental analysis, spectroscopy (IR, ^1^H and ^13^C NMR, and UV-Vis), molar conductivity measurement and cyclic voltammetry. Its crystal structure was determined by a single-crystal X-ray diffraction analysis.

### 2.2. Solid Studies

**Figure 2:**
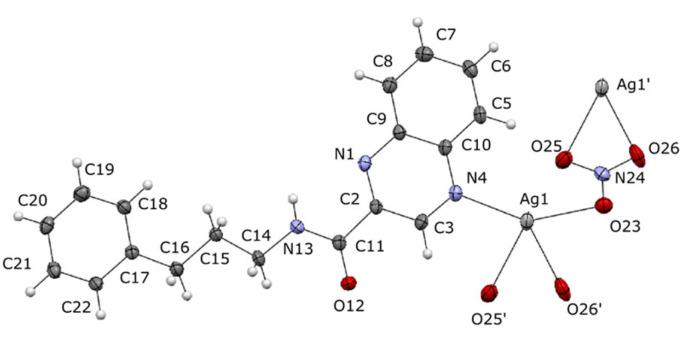
The asymmetric unit of the silver(I) complex with additional relevant atoms (Ag1’, O25’, O26’; symmetry code: *x*, 1 + *y*, *z*) showing the coordination environment of the central Ag(I) ion and indicating the polynuclear nature of the complex. Selected bond lengths around Ag1: Ag1−N4 2.251(2) Å, Ag1−O23 2.369(2) Å, Ag1−O25’ 2.654(2) Å, Ag1−O26’ 2.510(2) Å.

**Figure 3:**
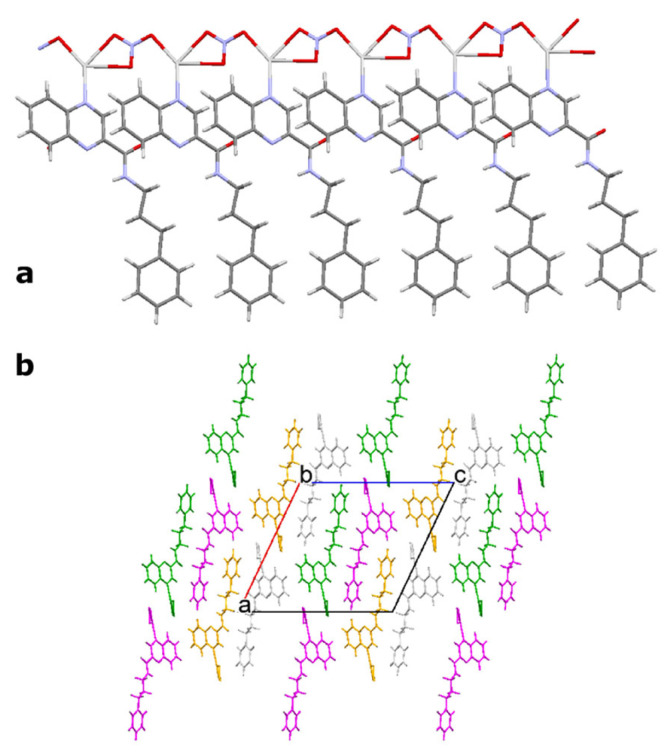
(**a**) The formation of AgNO_3_-spine to which pqx-2ca ligands are bound, and (**b**) the packing of 1D coordination chains to the crystal structure (a view along *b*-axis).

The asymmetric unit of the silver(I) complex is shown in Figure 2. It consists of silver(I) ion, one nitrate ion and one pqx-2ca ligand. The reaction between AgNO_3_ and pqx-2ca afforded a coordination polymer in which the Ag(I) ion lies in a highly distorted fourfold coordination environment with one nitrogen atom (N4) and three oxygen atoms (O23, O25’, O26’). The corresponding tau parameter values are 0.59 (τ4) and 0.57 (τ4’), respectively, confirming that the coordination geometry around the central ion deviates significantly from both ideal tetrahedral and ideal square-planar geometries. The nitrate ions act as bridges between the adjacent silver(I) ions, thus forming a spine to which the pqx-2ca ligands are additionally attached. This connectivity leads to the formation of 1D chains, which run along the crystallographic b-axis (Figure 3). Neighboring chains are connected only with weak intermolecular interactions, and no hydrogen bonds or π…π stacking interactions are present between them.

**Figure 4:**
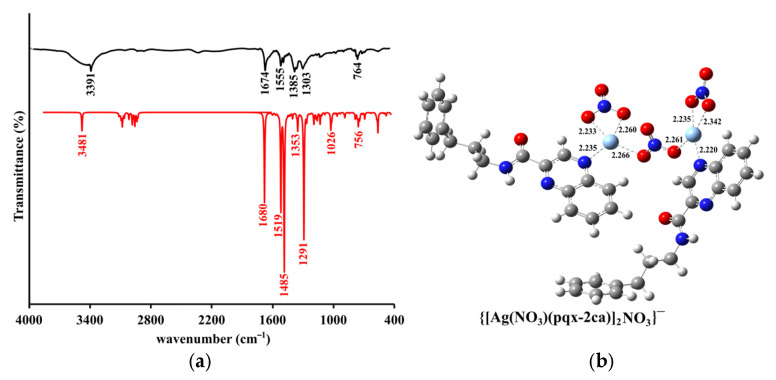
(**a**) Comparison of the experimental (black) and calculated (red) FT-IR spectra. (**b**) The optimized geometry of a selected fragment of the polymeric structure [Ag(NO_3_)(pqx-2ca)]_n_ in the gas phase. The calculations were performed using the B3LYP-D3BJ functional with the 6-311+G(d,p) basis set for all atoms, except Ag, for which the LANL2TZ(f) basis set was applied. Atom color code: Ag—light blue, C—gray, H—white, O—red, N—blue. Characteristic interatomic distances are indicated.

To make the vibrational analysis computationally tractable while still reflecting the local coordination environment present in the crystal, a conglomerate-type fragment was extracted from the polymeric chain and used as a representative model for the IR simulations. This truncated model preserves the key Ag–O/Ag–N connectivity and the immediate nitrate-bridged coordination motif that primarily governs the diagnostic vibrational modes, whereas long-range packing effects of the infinite polymer are not expected to significantly affect the positions of the main functional group bands. The IR spectrum of the synthesized silver(I) complex exhibits characteristic bands assigned to the amide and carbonyl groups of the pqx-2ca ligand, which are shifted towards lower wavenumbers compared to those of the uncoordinated ligand, thus confirming its coordination to the Ag(I) ion. Comparison of the experimental and theoretical IR spectra (Figure 4a) provides valuable insights into the vibrational features of the investigated silver(I) complex. Since the studied system exhibits a polymeric structure, the theoretical calculations were performed on a representative molecular fragment (Figure 4b), which adequately reproduces the main vibrational modes.

At higher wavenumbers, the bands observed at 3391 cm^−1^ (experimental) and 3481 cm^−1^ (theoretical) can be assigned to N–H stretching vibrations. In the calculated spectrum, additional contributions from C–H stretching modes are observed at slightly lower wavenumbers; however, in the experimental spectrum, these absorptions appear overlapped with the N–H stretching region, giving rise to a broad band.

In the carbonyl stretching region, strong absorptions at 1675 cm^−1^ (experimental) and 1680 cm^−1^ (theoretical) confirm the presence of C=O groups. Similarly, the bands at 1552 cm^−1^ (experimental) and 1519 cm^−1^ (theoretical) are attributed to a combination of N–C, N–H, and C–H stretching vibrations, further supporting ligand coordination through the nitrogen atom. The presence of intense bands at 1385 cm^−1^ (experimental) and 1353 cm^−1^ (theoretical), attributed to the asymmetric stretching vibrations of the NO_3_^−^ group, provides clear evidence of its coordination to the Ag(I) ion [24], fully consistent with the determined crystal structure. Finally, the low-frequency region reveals the influence of nitrate coordination, where the experimental band at 764 cm^−1^ and the calculated band at 756 cm^−1^ predominantly arise from O–N–O bending modes. This agreement between theory and experiment validates the structural model used in the calculations and confirms the proposed coordination environment around the silver(I) center.

### 2.3. Solution Studies

It should be emphasized that the solid-state crystal structure of a coordination compound does not necessarily reflect its predominant structure in solution. Differences between solid-state and solution-phase structures may arise from intermolecular interactions, crystal packing effects, and the absence of dynamic processes in the solid state. In contrast, coordination equilibria and partial dissociation may occur in solution. Such behavior is well documented for silver(I) complexes and related coordination systems [25]. In the present study, the single-crystal X-ray structure reveals a distorted coordination environment around the Ag(I) center, with notably different Ag–O bond lengths (Ag1–O23 2.369(2) Å vs. Ag1–O25′ 2.654(2) Å and Ag1–O26′ 2.510(2) Å), indicating weaker interactions associated with the longer Ag–O contacts. This structural asymmetry suggests that partial dissociation of the nitrate bridges is feasible under solution conditions, which is consistent with the spectroscopic and thermodynamic analyses discussed below.

A thermodynamic analysis of dissociation and substitution reactions was conducted using the Gibbs free energy of reaction (Δ*_r_G*), with calculations performed on a representative fragment of the polymeric structure (Table 1). Special attention was given to comparing the behavior of the complex in water, as a model of physiological conditions, and in DMSO, which was employed as a solvent for spectroscopic measurements (NMR and UV-Vis).

The obtained results demonstrate that the formation of the linear fragment [Ag(NO_3_)(pqx-2ca)] is thermodynamically favored in both solvents. The negative Gibbs free energy values (−0.31 kcal mol^−1^ in water and −0.22 kcal mol^−1^ in DMSO) indicate that the dissociation of the {[Ag(NO_3_)(pqx-2ca)]_2_NO_3_}^−^ complex, accompanied by the release of the nitrate anion into solution, is a spontaneous process. The equilibrium constants (*K* = 1.69 in water and *K* = 1.44 in DMSO) further confirm that the equilibria are slightly shifted toward the products, reflecting the relative stability of the linear complex fragments in both media. In contrast, substitution of the NO_3_^¯^ group by a DMSO molecule is not thermodynamically favored. The positive Δ*_r_G* (28.4 kcal mol^−1^) and the extremely low equilibrium constant (*K* ≈ 1.7 × 10^−21^) indicate that this process is both enthalpically and entropically unfavorable, and therefore negligible under experimental conditions. These results clearly highlight the role of the solvent: DMSO does not act as a substituent within the silver(I) coordination sphere, but rather as an aprotic polar medium suitable for solubilization and spectroscopic investigation. Based on these thermodynamic considerations, the linear [Ag(NO_3_)(pqx-2ca)] geometry was adopted as the most relevant structural model for describing the behavior of the complex in solution and for interpreting the corresponding spectroscopic data.

**Figure 5:**
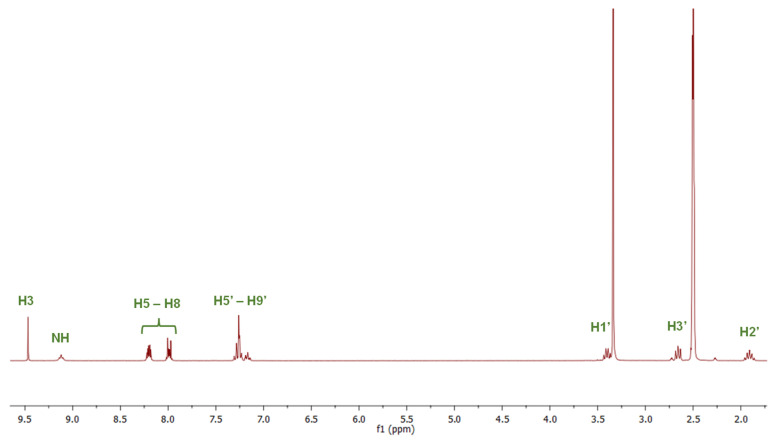
^1^H NMR spectrum of the silver(I) complex with pqx-2ca recorded in DMSO-*d*_6_ at room temperature.

Additional confirmation of the stability of the linear geometry of the complex in solution was obtained by comparing the simulated and experimental NMR spectra. ^1^H and ^13^C NMR spectra of the synthesized silver(I) complex were recorded in DMSO-*d*_6_ and contain the same number of signals as those of the uncoordinated ligand. All ^1^H resonances of the coordinated ligand in the complex are not significantly shifted compared to those of the free ligand, which is a characteristic spectroscopic property of silver(I) complexes in DMSO solution (Figure 5) [26].

Additional confirmation of the stability of the linear geometry of [Ag(NO_3_)(pqx-2ca)] in solution was obtained by comparing experimental and calculated NMR spectra (Table S1). In the ^1^H NMR spectrum, the signal for H3 (*δ* = 9.47 ppm) matches well with the theoretical value (*δ* = 9.83 ppm), while the aromatic region (*δ* = 8.23–7.96 and 7.31–7.12 ppm) shows good agreement with the calculated range (*δ* = 8.71–7.59 ppm). Similarly, in the ^13^C NMR spectrum, the carbonyl carbon C9 (*δ* = 163.1 ppm) corresponds closely to the calculated value (*δ* = 155.8 ppm), and both aromatic (*δ* = 145.0–125.0 vs. 143.0–126.3 ppm) and aliphatic carbons (*δ* = 39.0–30.0 vs. 42.8–37.3 ppm) are consistent. These results confirm that the linear geometry of the complex is favored in solution.

The stability of the synthesized silver(I) complex in a DMSO solution was monitored by UV-Vis spectroscopy over a 48 h period (Figure S1), a prerequisite for further biological evaluation. The positions of the absorption maxima do not show any significant change with time (only a decrease in the absorbance up to 12% was observed), suggesting that the pqx-2ca ligand remains coordinated to the Ag(I) ion throughout the tested period [27]. The experimental UV-Vis spectrum revealed two distinct absorption maxima at 316 nm and 327 nm, which correspond well with the theoretical predictions obtained using the TD-B3LYP-D3BJ/6-311++G(d,p) method (314 nm and 320 nm, respectively; Table 2). The minor deviations, within 2–7 nm, can be attributed to intrinsic limitations of the applied computational approach, particularly the use of implicit solvation models that neglect explicit solute–solvent interactions.

**Figure 6:**
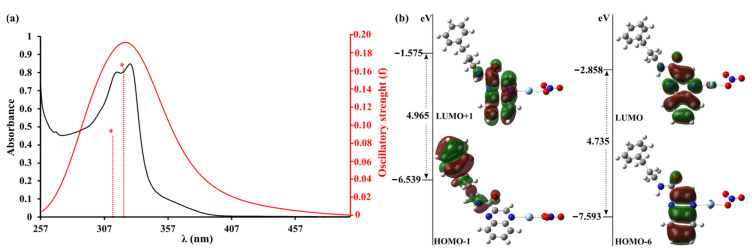
(**a**) Comparison of the experimental (black) and theoretical (red) UV-Vis spectra of the silver(I) complex with the pqx-2ca ligand. (**b**) The energy diagram of the HOMO and LUMO for the complex, illustrating the principal electronic transitions. Orbital energy levels are reported in eV, and the corresponding HOMO–LUMO energy gaps are also shown. The asterisk symbol (*) denotes the magnitude of the oscillator strength.

As illustrated in Figure 6a and summarized in Table 2, the absorption band centered at 316 nm arises mainly from the HOMO–1 → LUMO+1 transition (70%), with an oscillator strength of f = 0.069, indicating a moderate probability for this excitation. The second absorption band at 327 nm originates primarily from the HOMO–6 → LUMO transition (64%) and is characterized by a significantly higher oscillator strength (f = 0.190), which explains the experimentally observed stronger intensity of this band.

The good agreement between the experimental and theoretical spectra supports the validity of the chosen computational methodology for describing the electronic structure of the studied complex. Moreover, the orbital composition of the frontier molecular orbitals (Figure 6b) highlights the contribution of π → π* and n → π* transitions involving both ligand-centered and metal-to-ligand charge transfer (MLCT) characteristics. These transitions are consistent with the electronic properties of silver(I) complexes with nitrogen- and oxygen-donor ligands.

To further substantiate these spectroscopic findings, the molar conductivity of the synthesized complex was measured in DMSO solution. The obtained value, below 50 Ω^−1^cm^2^mol^−1^, indicates that the silver(I) complex behaves as a non-electrolyte and that the NO_3_^-^ and pqx-2ca ligands remain coordinated to the Ag(I) ion [28].

Moreover, the synthesized silver(I) complex was subjected to cyclic voltammetry (CV) studies to examine its electrochemical behavior, providing insights into its redox stability as well as its potential biological activity and mode of interaction with biomolecules [29]. Considering this, the stability of the complex was followed by CV in DMSO in the presence of 0.1 M tetrabutylammonium hexafluorophosphate (TBAHP) as a supporting electrolyte. The cyclic voltammograms of [Ag(NO_3_)(pqx-2ca)]_n_ and AgNO_3_ were recorded over a potential range from −2.0 to 2.0 V. As shown in Figure S2, one oxidation peak is observed, which can be attributed to the Ag(I) → Ag(II) process [30,31], while in the cathodic scan, two reduction peaks are detected, corresponding to Ag(II) → Ag(I) and Ag(I) → Ag(0) processes, respectively. Notably, the *I*_c,2_ peak is less pronounced in the voltammogram of the silver(I) complex compared to the corresponding salt, indicating that the reduction in the complex to the elemental silver is more difficult (Figure S2) [32,33].

### 2.4. BSA Binding Study

**Figure 7:**
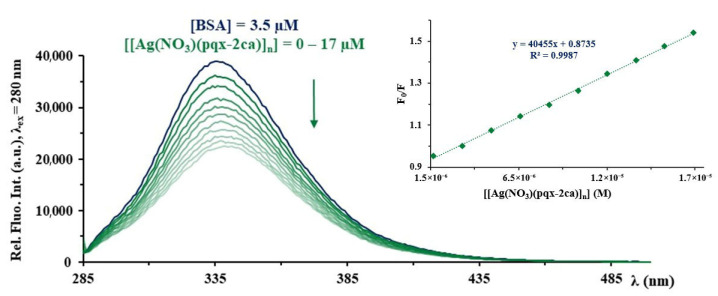
Fluorescence emission spectra of BSA in the absence and presence of increasing concentrations of the silver(I) complex. The arrow indicates the changes in fluorescence intensity with increasing complex concentration. Inserted graph: Stern–Volmer plots of *F_0_/F* vs. [compound].

In the present study, the possible binding affinity of the synthesized silver(I) complex toward serum albumin, a protein responsible for drug transport, was investigated by fluorescence emission spectroscopy [34] using bovine serum albumin (BSA) with two tryptophan residues responsible for its intrinsic fluorescence [35]. The emission spectra of BSA were recorded in the absence and presence of increasing amounts of the investigated silver(I) complex (Figure 7) and of the pqx-2ca ligand (Figure S3), using a BSA solution of constant concentration.

In both cases, a decrease in fluorescence intensity at 336 nm was observed, indicating that these compounds interact with the studied protein. To study the quenching mechanism, Stern-Volmer and Scatchard equations were applied and the obtained data (Stern-Volmer constants (*K_sv_*), quenching rate constants (*K_q_*), binding constants (*K_A_*), and the number of binding sites per BSA (*n*)) are presented in Table 3. The *K_A_* values indicate that the silver(I) complex and the pqx-2ca ligand exhibit similar affinity for binding to BSA and for transport to the cell [36], which is consistent with the calculated percentage of hypochromism and the *n* values for the compounds. At the same time, the *K_A_* values are not so high as to prevent the release of the compounds from BSA upon reaching the target cell [37]. The *K_q_* values for both compounds are much greater than 2 × 10^10^ M^−1^s^−1^, suggesting that quenching occurs predominantly via a static mechanism, resulting from the formation of a BSA–compound adduct [38,39].

Molecular docking simulations, performed after quantum-chemical optimization, provided insight into the interaction pattern of the [Ag(NO_3_)(pqx-2ca)] complex with BSA. This protein, a key serum transporter, consists of a 583-residue polypeptide organized into three large domains: domain I (residues 1–195), domain II (196–383), and domain III (384–583), each further subdivided into subdomains A and B [40,41,42]. The docking study indicated that the complex can be stably accommodated within all three domains of BSA (Table 4). Among these, domain III emerged as the most favorable site, characterized by the lowest inhibition constant and a predicted binding free energy of −11.74 kcal mol^−1^, reflecting strong affinity for this region. Domains I and II also supported robust interactions, confirming the broad binding potential of BSA toward the studied complex. The variations in calculated affinities are most likely associated with differences in structural topology and electronic distribution across the binding pockets. Collectively, these findings suggest that BSA can act as an efficient carrier, promoting both enhanced stability and facilitated transport of the complex under physiological conditions.

**Figure 8:**
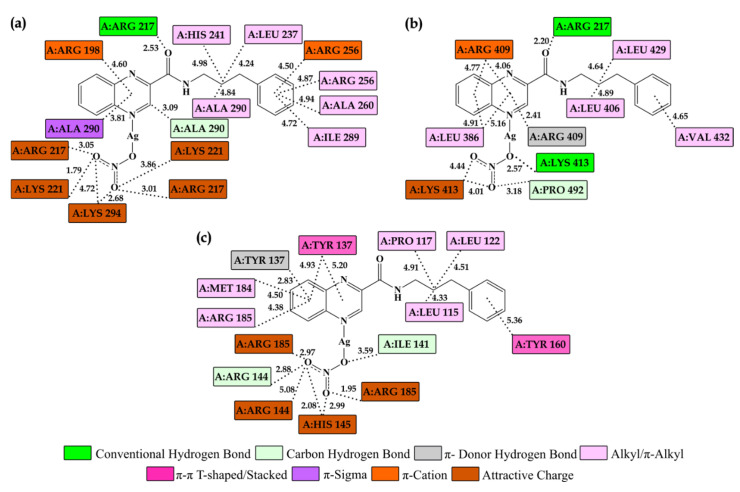
Noncovalent interactions of [Ag(NO_3_)(pqx-2ca)] with BSA illustrated at three binding domains: (**a**) site I (IIA), (**b**) site II (IIIA), and (**c**) site III (IB) shown in 2D molecular representation with interatomic distances (Å) and color-coded interaction types as indicated in the legend.

The interaction landscape of the complex within BSA comprises a diverse set of noncovalent forces that collectively contribute to its stabilization (Figure 8). Key contributors include conventional hydrogen bonds, hydrophobic alkyl and π–alkyl contacts, π–π stacking, and π–cation interactions, all reinforced by favorable electrostatic effects. The distribution of amino acid residues around the complex varies across domains I, II, and III, reflecting their distinct structural and electronic features. This diversity not only explains the observed differences in binding affinities but also highlights the ability of BSA to accommodate the silver(I) complex within multiple environments, ensuring both specificity and stability of the protein–complex association.

Hydrophobic contacts emerged as a defining feature of the interaction between the complex and amino acid residues positioned within all three binding domains. Residues such as alanine, leucine, and isoleucine were frequently engaged, with stabilization mainly achieved through alkyl and π–alkyl interactions that favorably anchor the complex inside the binding environment. Although these hydrophobic contributions were substantial, they did not act alone. A network of additional noncovalent forces was required to maintain the overall stability of the complex. Among these, π–sigma interactions, arising from the overlap between aromatic π clouds and neighboring σ bonds [43], were clearly evident at binding site I, particularly in association with residue A:ALA 290. π–π stacking effects further enhanced the conformational robustness of the complex, adopting both edge-to-face and offset parallel arrangements, depending on the steric and electronic parameters of the binding cavity [44,45]. At site III, these π–π interactions were primarily mediated by the aromatic rings of A:TYR 137 and A:TYR 160.

Another important contributor to the binding landscape was electrostatic interactions, which introduced a strong attractive component between the complex and positively charged residues located at different active sites. Through favorable spatial proximity, these charged interactions supported the embedding of the metal core and enhanced the overall stability. π–cation interactions represent electrostatic attractions established between positively charged species and π-electron–rich aromatic systems, whereas π–anion interactions arise from the association of negatively charged groups with electron-deficient π systems [46,47]. Such interactions were particularly evident in domains I and II, where π–cation contacts with A:ARG 198 and A:ARG 256 at site I, as well as A:ARG 409 in site II, played a dominant role in stabilizing the assembly.

The stability of the complex–protein assemblies is largely governed by the formation of classical hydrogen bonding interactions. These interactions were most prominent between A:ARG 217 and the carbonyl groups at sites I and II. In addition, a specific hydrogen bond was observed at site II between the oxygen atom of the nitro moiety and A:LYS 413. In addition to conventional hydrogen bonds, weaker but significant interactions, such as carbon-hydrogen bonds, were distributed across all three sites. Examples include A:ALA 290 at site I, A:PRO 492 at site II, and A:ILE 141 with A:ARG 144 at site III. π-donor hydrogen bonding was also reinforced by A:ARG 409 at site II and A:TYR 137 at site III, emphasizing the cooperative role of multiple bonding modes in ensuring complex stabilization and proper spatial positioning within the catalytic domain.

Taken together, the binding properties of the studied complex reflect a delicate equilibrium among hydrophobic forces, aromatic interactions, electrostatic contributions, and hydrogen bonding. Rather than being dominated by a single type of noncovalent contact, the stability and functional positioning of the complex are ensured through the synergistic interplay of multiple forces. This multifaceted stabilization mechanism not only illustrates the complexity of molecular recognition but also underscores the importance of cooperative effects in enabling strong and selective binding within a biologically relevant carrier protein such as BSA.

### 2.5. Lipophilicity Assay

Lipophilicity is a physicochemical property that plays an important role in ADMET (absorption, distribution, metabolism, elimination, and toxicology) parameters for all potential drug candidates. The most widely used method for determining the partition coefficient (log*P*), as a measure of lipophilicity, is the traditional shake-flask technique, which quantifies the distribution of a compound between a hydrophobic *n*-octanol phase and a hydrophilic water phase [48]. The calculated partition coefficient for the investigated silver(I) complex (log*P =* 0.32) falls within the range reported for various clinically used therapeutic agents, including metal complexes [49].

### 2.6. DNA Binding Experiments

The strong affinity of many heterocyclic aromatic compounds for DNA, either as intercalators or as minor- and major-groove binders that inhibit nucleic acid synthesis, has made them common agents in clinical therapy [50,51]. Compared with organic compounds, metal complexes offer a wide range of oxidation states, coordination numbers, and geometries, resulting in a virtually unlimited number of structures and enabling the design of geometries tailored for specific interactions [52]. The binding affinity of the pqx-2ca ligand and its silver(I) complex toward ct-DNA was investigated by fluorescence emission spectroscopy using a competitive binding approach with the intercalator ethidium bromide (EthBr) and with the minor groove binder Hoechst 33258 (2’-(4-hydroxyphenyl)-5-[5-(4-methylpiperazine-1-yl)benzimidazo-2-yl]-benzimidazole; Hoe). The emission spectra of ct-DNA-EthBr and ct-DNA-Hoe solutions, in which the ratio [ct-DNA]:[EthBr/Hoe] is 10:1, were recorded in the absence and presence of increasing concentrations of the investigated compounds.

As can be seen from Table 5, the silver(I) complex does not behave as an intercalative agent. This observation is consistent with the significantly lower percentage of hypochromism and binding constant compared with those of EthBr (Figure S4) [39]. In contrast, in the case of pqx-2ca, an increase in the fluorescence emission intensity of the ct-DNA-EthBr system was observed upon addition of the ligand.

**Figure 9:**
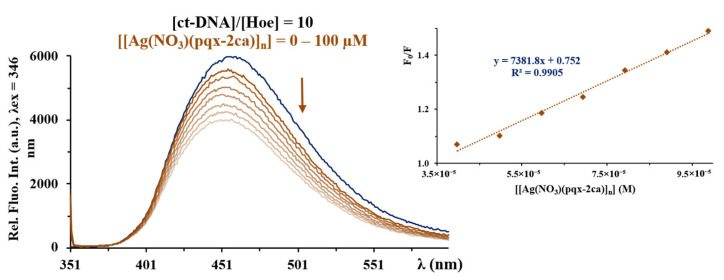
Fluorescence emission spectra of the Hoe-ct-DNA system in the absence and presence of increasing concentrations of the silver(I) complex. The arrow indicates the changes in fluorescence intensity with increasing complex concentration. Inserted graph: Stern–Volmer plots of *F_0_/F* vs. [compound].

On the other hand, titration of the ct-DNA–Hoe system with the investigated compounds resulted in a significant decrease in fluorescence emission intensity upon increasing compound concentrations (Figure 9 and Figure S5). This effect can be attributed to the ability of the investigated compounds to displace Hoe from the minor groove of the DNA duplex [53].

As can be seen from Table 5, the *K*_A_ values for binding of the investigated compounds to the ct-DNA-Hoe system are much higher than that for Hoe itself (*K*_A_ = 2 × 10^6^ M^−1^), indicating their significantly stronger interaction with ct-DNA via minor groove binding. This conclusion is further supported by the calculated Stern-Volmer constants. Moreover, the *K_q_* values for both investigated compounds suggest a static quenching mechanism resulting from their interaction with the ct-DNA-Hoe system (Table 5) [39,54].

To further examine the binding behavior of the silver(I) complex with nucleic acids, two representative DNA models, 1BNA and 1Z3F, were selected for docking analysis. The 1BNA structure corresponds to the canonical B-DNA dodecamer, d(CGCGAATTCGCG)_2_, which serves as a standard reference for the right-handed double helix. In contrast, 1Z3F represents a hexanucleotide duplex, d(CGATCG)_2_, co-crystallized with the intercalating anticancer agent ellipticine, making it a suitable model for evaluating intercalative binding modes. Docking results revealed a stronger preference of the silver(I) complex for the canonical B-DNA duplex, which exhibited more favorable binding energies and interaction profiles compared with the intercalative DNA model (Table 6). Within the B-DNA grooves, stabilization was largely mediated by hydrogen bonds and π–π stacking contacts, aligning the complex with the helical framework. In contrast, binding to the 1Z3F structure was weaker and less stable.

**Figure 10:**
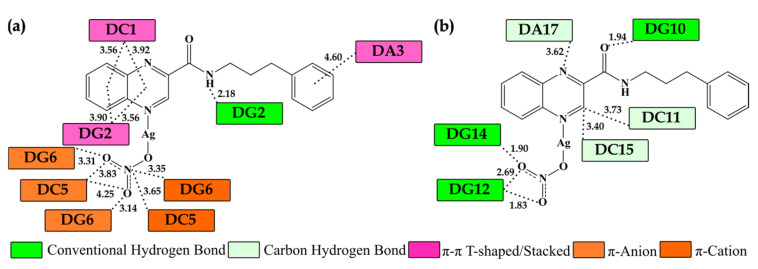
Noncovalent interactions between the [Ag(NO_3_)(pqx-2ca)] complex and the binding regions of (**a**) the hexanucleotide d(CGATCG)_2_ (PDB ID: 1Z3F), and (**b**) canonical B-DNA (PDB ID: 1BNA), shown in 2D molecular representation with interatomic distances (Å) and color-coded interaction types as indicated in the legend. Labels used for the interacting nucleosides are as follows: DC—deoxycytidine, DA—deoxyadenosine, and DG—deoxyguanosine.

Figure 10 provides a visual representation of the interactions of the [Ag(NO_3_)(pqx-2ca)] complex with DNA. In the B-DNA model, the complex aligns within the grooves of the helix, forming stabilizing hydrogen bonds with nucleobase functional groups and establishing π–π stacking interactions with adjacent base pairs. This orientation promotes efficient accommodation of the complex and contributes to its structural stability within the helical framework. In contrast, the 1Z3F duplex reveals a different binding mode, where the complex intercalates between successive base pairs. This positioning strengthens π–π stacking forces, while additional contributions from hydrophobic alkyl/π–alkyl interactions and localized hydrogen bonding further stabilize the intercalated complex.

The silver(I) complex establishes a diverse network of noncovalent interactions with nucleoside residues, in both the canonical B-DNA and the intercalative 1Z3F duplex. Among the most prominent stabilizing elements are hydrogen bonds. In the 1Z3F structure, the complex forms a distinct hydrogen bond with nucleoside DG2, while in B-DNA it engages the polarized oxygen atom of the nitro moiety with DG12 and DG14, and the carbonyl oxygen with DG10. Additional carbon–hydrogen contacts contribute to the stability of the B-DNA complex, particularly through interactions with DC11, DC15, and DA17.

Hydrophobic interactions also reinforce the binding framework. In the intercalative 1Z3F model, π–π stacking emerges as a dominant interaction, especially between the aromatic moieties of the complex and nucleosides DC1, DG2, and DA3, creating a compact and stabilized assembly through planar overlap of aromatic systems.

Electrostatic forces also play a defining role in shaping the binding profile. Within 1Z3F, π–anion interactions arise from the complementary alignment of negatively charged oxygen atoms of the nitro substituent with nucleosides DC5 and DG6, while the nitrogen atom of the same group simultaneously engages in π–cation interactions with the same nucleosides. These cooperative charge-based interactions not only enhance stability but also introduce a level of specificity, reflecting a balance between electronic complementarity and structural adaptability in DNA–metal complex recognition.

Overall, the interaction analysis reveals that [Ag(NO_3_)(pqx-2ca)] binds DNA through a synergistic network of hydrogen bonding, hydrophobic, and electrostatic contacts. These complementary forces ensure both stability and specificity of binding, underscoring the adaptability of the silver(I) complex toward distinct DNA conformations.

### 2.7. Antimycobacterial Activity of the Silver(I) Complex

A recent review summarized findings from numerous studies evaluating the antimycobacterial activity of various metal complexes against *M. tuberculosis* [55]. A comprehensive analysis of the reported data indicated that silver(I) complexes generally exhibit notable activity, with average MIC values below 30 μg mL^−1^. In addition, the activity of three silver(I) complexes containing *N*′-[(*E*)-(pyridine-2-ylmethylene)pyrazine-2-carbohydrazide] was investigated, showing good activity against *M. tuberculosis* H37Rv, with MIC values below 10 μg mL^−1^ [56].

Accordingly, we assessed the antimycobacterial potential of the synthesized silver(I) complex with the pqx-2ca ligand against several mycobacterial strains. The results demonstrated that the silver(I) complex exhibited markedly enhanced activity compared to the free ligand, with MIC values ranging from 1.98 to 15.625 µg mL^−1^ (4.3 to 33.9 µM) (Table 7). The strongest activity was observed against *M. smegmatis* and *M. aurum*, with MIC values of 1.98 µg mL^−1^ (4.3 µM). The observed MIC values of the synthesized complex against *M. avium* and *M. kansasii* are slightly lower than those reported for previously evaluated polymeric, water-soluble silver(I) complexes containing α-hydroxycarboxylic acids (mandelic, glycolic, tartaric and malic) [57]. Furthermore, the MIC value obtained for the [Ag(NO_3_)(pqx-2ca)]ₙ complex against *M. avium* suggests higher activity compared with isoniazid, a standard antitubercular drug [58].

### 2.8. Docking-Based Insights into the Interaction of the Complex with Mycolic Acid Pathway Enzymes

To further evaluate the potential of the silver(I) complex with the pqx-2ca ligand as a bioactive scaffold, its interactions with key mycobacterial enzymes involved in cell wall biosynthesis were investigated. Against the InhA, the complex exhibited a binding affinity nearly comparable to that of the reference inhibitor, with a calculated Δ*G_bind_* of −7.95 kcal mol^−1^ (Table 8). Although the overall free energies were nearly identical, a closer inspection of the individual energy components revealed subtle distinctions, suggesting slight variations in the underlying interaction patterns. These results indicate that the silver(I) complex can adopt a binding mode within InhA that is energetically competitive with its established inhibitor. In the case of MmpL3, however, the docking results showed more pronounced differences. The complex achieved a binding free energy of −9.10 kcal mol^−1^, associated with a submicromolar inhibition constant, whereas the reference inhibitor exhibited slightly stronger affinity. Despite this, the complex still displayed considerable binding strength, indicating that MmpL3 remains a viable target for its interaction.

**Figure 11:**
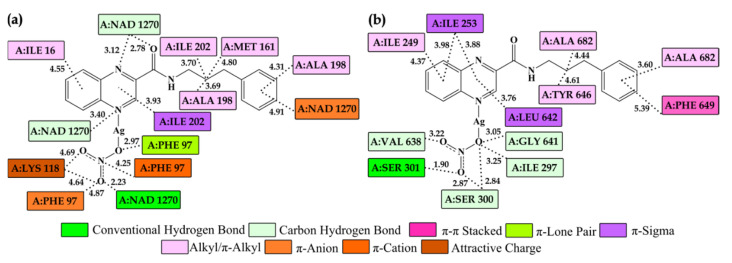
Noncovalent interactions between the [Ag(NO_3_)(pqx-2ca)] complex and the protein targets (**a**) InhA and (**b**) MmpL3, shown in 2D molecular representation with interatomic distances (Å) and color-coded interaction types as indicated in the legend.

The interaction schemes in Figure 11 illustrate that the complex engages in a diverse set of stabilizing contacts within both enzyme pockets. In the case of InhA, the binding mode is reinforced by a combination of hydrogen bonds and aromatic interactions, including π–π stacking and π–cation contacts with key residues. In contrast, within the MmpL3 cavity, the complex is stabilized through a more extensive hydrogen-bonding network, which is further strengthened by π–π stacking effects. Collectively, these patterns indicate that hydrophobic and aromatic forces contribute more significantly to the stabilization of InhA, whereas polar interactions dominate in MmpL3, consistent with the observed binding affinities.

Hydrogen bonding plays a crucial role in reinforcing the structural framework of the studied complex. In InhA, a prominent polar interaction is established with residue A:NAD 1270, while in MmpL3, a distinct hydrogen bond is formed with A:SER 301. Beyond classical hydrogen bonds, additional stabilizing forces arise from weaker yet meaningful carbon-hydrogen interactions, including those with A:NAD 1270 in InhA, and with A:ILE 297, A:SER 300, A:VAL 638, and A:GLY 641 in MmpL3.

Hydrophobic interactions emerged as another defining feature of the binding profile, prominently involving residues such as alanine and isoleucine. Stabilization is primarily achieved through alkyl and π–alkyl contacts, which secure the complex within the enzyme cavities. Within this category, π–sigma interactions were particularly evident with residue A:ILE 202 in InhA, as well as with A:ILE 253 and A:LEU 642 in MmpL3. Furthermore, π–π stacking interactions contributed to stabilization, especially between the aromatic moiety of the complex and residue A:PHE 649 in MmpL3.

Electrostatic interactions added an additional layer of binding stability. Notably, π–cation interactions were formed between the nitrogen atom of the nitro substituent and residue A:PHE 97, while π–anion contacts involved the oxygen atom of the same group and the same residue. Additional attractive charge interactions were also observed with A:LYS 118, further supporting the stability and specificity of the enzyme–complex systems. 

The interaction pattern observed for the [Ag(NO_3_)(pqx-2ca)] complex within InhA and MmpL3 underscores the importance of complementary noncovalent forces in shaping enzyme–complex recognition. The combination of interactions generates a stabilization network that resembles, though does not identically replicate, the interactions formed by reference inhibitors. This interplay highlights the adaptability of the silver(I) complex to structurally distinct enzymatic pockets and points toward its potential as a promising scaffold for the development of novel antimycobacterial therapeutics.

## 3. Materials and Methods

### 3.1. Materials and Instruments

Silver(I) nitrate, ethanol, dimethyl sulfoxide (DMSO), deuterated dimethyl sulfoxide (DMSO-*d*_6_), phosphate-buffered saline (PBS), bovine serum albumin (BSA), calf thymus DNA (ct-DNA), ethidium bromide (EthBr) and (2’-(4-hydroxyphenyl)-5-[5-(4-methylpiperazine-1-yl)benzimidazo-2-yl]-benzimidazole; Hoechst 33258; Hoe) were purchased from the Sigma-Aldrich (Schnelldorf, Germany). All chemicals were of analytical reagent grade and used without further purification.

Elemental microanalyses of the synthesized silver(I) complex for carbon, hydrogen, and nitrogen were performed by the Department of Science, Institute for Information Technologies Kragujevac, University of Kragujevac. The IR spectra were recorded on a Perkin Elmer Spectrum 2 spectrometer using the KBr pellet technique over the wavenumber range of 4000–450 cm^−1^ (abbreviations used: br for broad, vs for very strong, s for strong, m for medium, w for weak). The NMR (^1^H and ^13^C) spectra of the *N*-(3’-phenylpropyl)quinoxaline-2-carboxamide and its silver(I) complex were recorded at room temperature on a Varian Gemini 2000 spectrometer (^1^H at 200 MHz, ^13^C at 50 MHz, Varian, Inc., Palo Alto, CA, USA). 5.0 mg of each compound was dissolved in 0.6 mL of DMSO-*d*_6_ and transferred into a 5 mm NMR tube. Chemical shifts, *δ*, are reported in ppm (parts per million), and scalar couplings (*J*) are reported in Hertz. Chemical shifts were calibrated relative to those of the solvent. Peak multiplicities are denoted as follows: *s* = singlet, *dd* = doublet of doublets, *t* = triplet, and *m* = multiplet. The UV-Vis spectra of the *N*-(3’-phenylpropyl)quinoxaline-2-carboxamide and its silver(I) complex were recorded over the wavelength range of 1100–200 nm on a Shimadzu UV-1800 spectrophotometer at room temperature after dissolution in DMSO. The concentration of the solutions in DMSO used for UV-Vis measurements was 1.15 × 10^−4^ and 1.10 × 10^−4^ M, respectively. To investigate the solution stability of the synthesized complex, UV–Vis spectra were recorded again 24 h and 48 h after dissolution. The solution behavior of the silver(I) complex was further studied by measuring its molar conductivity using a digital conductivity meter (Crison Multimeter MM 41) after dissolution in DMSO at room temperature. The concentration of the solution of silver(I) complex in DMSO used for these measurements was 1.0 × 10^−3^ M. Cyclic voltammetry (CV) measurements were performed using a potentiostat/galvanostat AutoLab PGSTAT204 (Utrecht, The Netherlands). The cell (5.0 mL) consisted of a three-electrode system, a glassy carbon (GC) electrode as a working electrode, Ag/AgCl (saturated KCl) as a reference electrode, and a platinum wire as a counter electrode. All reported potentials are referred to the Ag/AgCl (saturated KCl) reference electrode. The electrode surface was renewed before every measurement by polishing with Al_2_O_3_ micro-powder and with a piece of cotton due to the strong adsorption of the complexes. The concentration of the solution of the complex in DMSO used for these measurements was 1.0 × 10^−3^ M. Emission spectra for BSA and DNA interactions of the silver(I) complex and the ligand used for its synthesis were recorded using a Jasco FP-6600 spectrophotometer.

### 3.2. Preparation of N-(3’-phenylpropyl)quinoxaline-2-carboxamide

The *N*-(3’-phenylpropyl)quinoxaline-2-carboxamide (pqx-2ca), used as a ligand for the synthesis of silver(I) complex, was prepared following a previously described method by reacting quinoxaline 2-carboxylic acid, activated with oxalyl chloride, with the corresponding amine [19]. The purity of the synthesized compound was determined by elemental analysis and NMR spectroscopy.

pqx-2ca: MW = 291.35 g mol^−1^. IR (KBr, *ν*, cm^−1^): 3397s (*ν*(N–H)), 3066w, 3020w (*ν*(C_ar_–H)), 2960w, 2935m (*ν*(C–H)), 1679vs (*ν*(C=O), 1605w, 1574w, 1554s, 1523s, 1492m, 1456w, 1438w, 1408w, 1366w (*ν*_as_(C_ar_=C_ar_) and *ν*_as_(C_ar_=N)), 749m (*γ*(C_ar_–H)). ^1^H NMR (200 MHz, DMSO-*d*_6_): *δ* = 9.47 (*s*, H3); 9.12 (*t*, NH); 8.24–7.93 (*m*, H5–H8); 7.34–7.11 (*m*, H5’–H9’); 3.40 (*dd*, *J* = 13.6; 6.9 Hz; H1’); 2.66 (*m*, H3’); 1.91 (*m*, H2’) ppm. ^13^C NMR (50 MHz, DMSO-*d*_6_): *δ* = 163.1 (C9); 145.0–125.0 (A_r_-C); 39.0–30.0 (C1’–C3’) ppm.

### 3.3. Preparation of the Silver(I) Complex

The silver(I) complex [Ag(NO_3_)(pqx-2ca)]_n_ was synthesized following a modified procedure previously reported for the preparation of silver(I) complexes with pyridazine, pyrimidine, pyrazine, quinoxaline and phenazine [59]. A solution of 1.0 mmol of AgNO_3_ (169.9 mg) in 5.0 mL of ethanol (96%) was added dropwise under stirring to a solution containing an equimolar amount of the pqx-2ca (291.6 mg) in 5.0 mL of ethanol (96%). The reaction mixture was stirred at ambient temperature in the dark for 3 h. No visible color change or precipitation was observed during this period. The resulting clear solution was then left to slowly evaporate at room temperature in the dark. After 3 days, colorless crystals of the complex formed, which were filtered and dried in the dark at ambient temperature. Yield: 272.1 mg (59%).

Anal. Calc. for [Ag(NO_3_)(pqx-2ca)]_n_ (C_18_H_17_AgN_4_O_4_; MW = 461.22 g mol^−1^): C, 46.87; H, 3.72; N, 12.15. found: C, 46.49; H, 3.59; N, 12.01%. IR (KBr, *ν*, cm^−1^): 3391vs (*ν*(N–H)), 3055w (*ν*(C_ar_–H)), 2932w, 2870w (*ν*(C–H)), 1675vs (*ν*(C=O), 1581w, 1552w, 1541w, 1520s, 1495m, 1473w, 1453w (*ν*_as_(C_ar_=C_ar_) and *ν*_as_(C_ar_=N)), 1385vs, 1365s (*ν*_as_(NO_3_)), 764m (*γ*(C_ar_–H)). ^1^H NMR (200 MHz, DMSO-*d*_6_): *δ* = 9.47 (*s*, H3); 9.12 (*t*, NH); 8.23–7.96 (*m*, H5–H8); 7.31–7.12 (*m*, H5’–H9’); 3.40 (*dd*, J = 13.6; 6.7 Hz; H1’); 2.66 (*m*, H3’); 1.91 (*m*, H2’) ppm. ^13^C NMR (50 MHz, DMSO-*d*_6_): *δ* = 163.1 (C9); 145.0–125.0 (A_r_-C); 39.0–30.0 (C1’–C3’) ppm. UV-Vis (DMSO, *λ*_max_, nm): 316 (*ε* = 7.10 × 10^3^ M^−1^cm^−1^), 327 (*ε* = 7.50 × 10^3^ M^−1^cm^−1^). *Λ*_M_ (DMSO): 37.5 Ω^−1^cm^2^ mol^−1^.

### 3.4. Crystallographic Data Collection and Refinement of the Structures

For X-ray structural analysis, a suitable single crystal was surrounded by silicon grease, mounted onto the tip of a glass fiber and transferred to the goniometer head in a liquid nitrogen cryostream (T = 150 K). Data were collected on a SuperNova diffractometer equipped with an Atlas detector using CrysAlis software and monochromated Mo Kα radiation (0.71073 Å) [60]. The initial structural model was obtained via a dual-space algorithm using the SHELXT structure solution program [61]. A full-matrix least-squares refinement on F^2^ magnitudes with anisotropic displacement parameters for all nonhydrogen atoms was performed using SHELXL-2018/3 [62]. All H atoms were initially located in difference Fourier maps; those residing on C-atoms were further treated as riding on their parent atoms with C(aromatic)−H and C(methylene)-H distances of 0.95 and 0.99 Å, respectively. On the other hand, the hydrogen atom bonded to nitrogen atoms was found in the difference Fourier maps and refined isotropically without constraints or restraints. Details on crystal data, data collection and structure refinement are given in Table S2. Figures depicting the structures were prepared with Mercury [63].

### 3.5. BSA Binding Experiments

The protein binding study was performed by tryptophan fluorescence quenching experiments using BSA (3.5 μM) in phosphate-buffered solution (pH 7.4). The quenching of the emission intensity of the tryptophan residues of BSA at 336 nm was monitored at increasing concentrations of the pqx-2ca ligand and the silver(I) complex (up to 17.0 μM). Fluorescence spectra were recorded over the range of 285–500 nm with an excitation wavelength of 280 nm. Stern–Volmer constants (*K_sv_*) were calculated according to the Stern–Volmer equation [38,64]:

F_0_/F = 1 + *K_q_τ*_0_[complex] = 1 + *K_sv_*[complex]

where F_0_ and F are the fluorescence intensities in the absence and presence of the synthesized complex, respectively, *K_q_* is the bimolecular quenching constant, and *τ*_0_ (10^−8^ s) is the average fluorescence lifetime of the fluorophore in the absence of quencher. Binding constants and the apparent binding sites were calculated using the following equation [38,64]:

log(F_0_ − F)/F = log*K_A_* + *n*log[complex]

where *K_A_* represents the binding constant of the complex with BSA, and *n* is the apparent number of binding sites per BSA molecule.

### 3.6. Lipophilicity Assay

The lipophilicity of the silver(I) complex was determined using the flask-shaking method [48]. The complex was dissolved in DMSO and added to a water/*n*-octanol system. This mixture was vortexed for 1 h at 25 °C to allow partitioning and then left to stand for 24 h to achieve complete phase separation. The concentration of the complex in both phases was determined by measuring absorbance values using previously determined calibration curves. The log*P* values were calculated according to the following equation:

log*P* = log(c_0_ /c_w_)

where c_0_ and c_w_ are the concentrations of the complex in the n-octanol and water phases, respectively.

### 3.7. ct-DNA Binding Experiments

The silver(I) complex and pqx-2ca were dissolved in DMSO (10 mM). A stock solution of ct-DNA was prepared by dissolving the solid substance in PBS. The concentration of the ct-DNA solution was determined from UV absorbance at 258 nm using the molar extinction coefficient *ε* = 6.6 × 10^3^ M^−1^cm^−1^ [65]. Stock solutions of EthBr and Hoe were freshly prepared in DMSO (10 mM) and stored at 4 °C prior to use.

Competitive binding studies were performed at pH 7.4 in PBS, whereas the ratio [ct-DNA]:[EthBr/Hoe] was 10:1, while the concentration of the complex gradually increased (up to 100 μM). The spectra for the competitive interaction between EthBr and the silver(I) complex toward ct-DNA were recorded over the range of 525–750 nm, with an excitation wavelength of 520 nm. For Hoe, the spectra were recorded over 351–750 nm, with an excitation wavelength of 346 nm. The corresponding binding constants (*K_A_*) and apparent number of binding sites (*n*) were calculated as described previously [38,64].

### 3.8. Antimycobacterial Activity

#### 3.8.1. Mycobacterial Strains

The antimycobacterial assay was performed using an avirulent strain of *Mycobacterium tuberculosis* H37Ra ITM-M006710 (ATCC 9431) from Belgian Co-ordinated Collections of Micro-organisms (Antwerp, Belgium), rapidly growing *Mycolicibacterium smegmatis* DSM 43465 (ATCC 607) and *Mycolicibacterium aurum* DSM 43999 (ATCC 23366), and nontuberculous mycobacteria, namely *Mycobacterium avium* subsp. *avium* DSM 44156 (ATCC 25291) and *Mycobacterium kansasii* DSM 44162 (ATCC 12478) from German Collection of Microorganisms and Cell Cultures (Braunschweig, Germany).

#### 3.8.2. Experimental

The technique used for activity determination was the microdilution broth panel method using 96-well microtitration plates (Gamedium, Czech Republic) [66,67]. The liquid culture medium used in all assays was Middlebrook 7H9 broth (Merck, Darmstadt, Germany) enriched with 0.4% glycerol (*v*/*v*) (Merck, Darmstadt, Germany) and 10% Middlebrook OADC growth supplement (Himedia, Mumbai, India). Mycobacterial strains were cultured on supplemented Middlebrook 7H9 agar at −37 °C in static, dark humid atmosphere. The final density of starting inoculum was adjusted to 1.0 according to the McFarland scale and diluted in the ratio of either 1:20 (for rapidly growing mycobacteria) or 1:10 (for slow growing mycobacteria) with broth.

Tested compounds were dissolved in DMSO (Merck, Darmstadt, Germany), then Middlebrook broth was added to obtain a concentration of 2000 µg mL^−1^. The standards used for internal quality control were isoniazid (INH), rifampicin (RIF), and ciprofloxacin (CIP) (Merck, Darmstadt, Germany). Final concentrations were reached by binary dilution and addition of mycobacterial suspension and were set as 500, 250, 125, 62.5, 31.25, 15.625, 7.81, and 3.91 µg mL^−1^. The final concentration of DMSO did not exceed 2.5% (*v*/*v*) and did not affect the growth of all strains. Positive (broth, DMSO, bacteria) and negative (broth, DMSO) growth controls were included. The addition of 20 µL 0.01% solution of resazurin sodium salt (Thermo Scientific Chemicals, Waltham, Massachusetts, USA) followed after 48 h of incubation for *M. smegmatis*, after 72 h for *M. aurum*, after 96 h for *M. avium* and *M. kansasii*, and after 120 h for *M. tuberculosis*. Microtitration panels were then incubated for an additional 2.5 h to determine the activity against *M. smegmatis*, 4 h for *M. aurum*, 6 h for *M. avium* and *M. kansasii*, and 18 h for *M. tuberculosis*. Antimycobacterial activity was expressed as minimum inhibition concentration (MIC), and the value was read on the basis of stain color change (blue color—active compound; pink color—inactive compound). All experiments were performed in duplicates.

### 3.9. Computational Methodology

#### 3.9.1. Theoretical Approach and Spectral Simulation

To complement the experimental results and gain a deeper understanding of the molecular behavior, quantum chemical calculations were performed using the Gaussian16 program package [68]. Geometry optimization of the [Ag(NO_3_)(pqx-2ca)] complex was carried out using the B3LYP-D3BJ functional in combination with the 6-311+G(d,p) basis set for all atoms, while the LANL2TZ(f) effective core potential was applied for silver [69]. Solvent effects were included through the conductor-like polarizable continuum model (CPCM) [70], which was included in both geometry optimization and single-point energy calculations to mimic the dielectric environment of the investigated solvents. Theoretical ^1^H and ^13^C NMR chemical shifts were obtained using the Gauge-Independent Atomic Orbital (GIAO) method [71,72] with tetramethylsilane (TMS) used as the reference standard. In addition, UV-Vis spectra were simulated by time-dependent density functional theory (TD-DFT), employing DMSO as the solvent for the silver(I) complex in accordance with the experimental setup. To account for the polymeric nature of the complex in the solid state and its partial dissociation in solution, different representative fragments were employed for spectral simulations. While IR spectra were modeled using a fragment preserving the local polymeric coordination motif, NMR and UV-Vis simulations were performed on the thermodynamically favored linear species relevant under solution conditions. This integrated computational approach (combining structural optimization, implicit solvation, and spectroscopic simulations) enabled a consistent comparison with experimental observations and provided valuable insights into the structural and electronic properties of the studied complex.

#### 3.9.2. Silver(I) Complex as Biomolecular Binder: An In Silico Study

Computational chemistry provides a powerful framework for understanding the behavior of metal complexes, offering both structural and energetic insights into their interactions with biological macromolecules. In the present study, particular attention was devoted to the silver(I) complex [Ag(NO_3_)(pqx-2ca)], whose binding preferences toward proteins and nucleic acids were explored using an integrated approach combining quantum-chemical calculations and molecular docking. The selected receptors included bovine serum albumin (BSA, PDB ID: 4F5S) [73], the B-DNA duplex (PDB ID: 1BNA) [74], and the intercalated DNA hexamer d(CGATCG)_2_ (PDB ID: 1Z3F) [75], enoyl-acyl carrier protein reductase from *Mycobacterium tuberculosis* InhA (PDB ID: 2X22) [76], and the mycolic acid transporter MmpL3 from *Mycobacterium smegmatis* (PDB ID: 6AJG) [77]. All crystal structures were retrieved from the RCSB Protein Data Bank and prepared using BIOVIA Discovery Studio 4.0 [78], with crystallographic water molecules, co-bound ligands, and non-essential heteroatoms removed. Docking simulations were performed using AutoDock Tools 1.5.7 and AutoDock 4.2.6 [79]. The Lamarckian Genetic Algorithm (LGA) [80] was employed, allowing full ligand flexibility while keeping the receptor rigid. Grid boxes were defined around the major binding regions of each target. Docking grids were carefully defined to encompass key binding regions for each macromolecular target. For BSA, three binding sites were examined: site I (IIA) at −4.80 × 30.50 × 101.01 Å, site II (IIIA) at 10.91 × 16.30 × 119.72 Å, and site III (IB) at 19.86 × 33.53 × 97.92 Å, each with grid dimensions of 60 × 60 × 60 Å and a spacing of 0.375 Å. For the B-DNA duplex, the docking grid was set to 60 × 74 × 120 Å and centered at 15.81 × 21.31 × 9.88 Å. For InhA, the grid was centered at −21.445 × −32.127 × 13.779 Å with box dimensions of 40 × 40 × 40 Å, while for MmpL3, the grid was centered at 101.8019 × 17.2934 × 32.6825 Å with dimensions of 69 × 69 × 69 Å. The genetic algorithm was executed with a population size of 150, 250,000 energy evaluations, and 27,000 generations, with mutation and crossover rates of 0.02 and 0.8, respectively. This computational strategy enabled a comprehensive exploration of the interaction patterns of the silver(I) complex with diverse biomolecular systems. The resulting thermodynamic and structural insights complement the experimental findings and provide a deeper understanding of the potential bioactivity and therapeutic relevance of the investigated complex.

## 4. Conclusions

In this work, a new polynuclear silver(I) complex, [Ag(NO_3_)(pqx-2ca)]ₙ, derived from the *N*-(3′-phenylpropyl)quinoxaline-2-carboxamide (pqx-2ca) ligand, was synthesized and comprehensively characterized using crystallographic, spectroscopic, electrochemical, and computational approaches. Single-crystal X-ray diffraction revealed a polymeric structure in which pqx-2ca coordinates Ag(I) in a monodentate manner, while nitrate acts as a bridging ligand, giving rise to 1D coordination chains. Thermodynamic analyses demonstrated that partial dissociation to the linear [Ag(NO_3_)(pqx-2ca)] species is slightly favored in both aqueous media and DMSO, whereas substitution of nitrate by DMSO is strongly disfavored, consistent with the observed non-electrolyte behavior in solution.

When compared to the free ligand, the complex exhibits markedly enhanced in vitro antimycobacterial activity, with MIC values in the range of 1.98 to 15.625 µg mL^−1^ (4.3 to 33.9 µM) against the avirulent *Mtb* H37Ra, the fast-growing *M. smegmatis* and *M. aurum*, as well as the nontuberculous *M. avium* and *M. kansasii* strains. These strains were deliberately selected to represent different growth rates, cell-wall compositions, and pathogenic relevance, thereby enabling a broader and more informative assessment of antimycobacterial potential. The observed MIC values demonstrate that the complex is particularly effective against fast-growing species, while retaining notable activity toward slow-growing and pathogenic mycobacteria.

Protein and DNA interaction studies revealed moderate and reversible binding to bovine serum albumin, compatible with serum transport and release, as well as a clear preference for DNA minor-groove binding rather than intercalation. Molecular docking further supported these experimental findings and indicated energetically favorable binding of the complex to key enzymes of the mycolic-acid biosynthetic pathway, namely InhA and MmpL3, identifying plausible molecular targets that may contribute to the observed antimycobacterial activity.

Overall, the convergence of crystallography, spectroscopy, computation, and bioassays establishes [Ag(NO_3_)(pqx-2ca)]ₙ as a structurally well-defined silver(I) assembly with promising antimycobacterial potency. Taken together, the obtained experimental and computational results confirm the initial hypothesis that coordination of pqx-2ca to Ag(I) leads to a complex with enhanced antimycobacterial activity, supported by dual biomolecular interactions with DNA and proteins, as well as energetically favorable binding to key mycobacterial targets. Future efforts should focus on tuning the first coordination sphere (e.g., non-labile co-ligands, alternative counter-anions), modulating lipophilicity and serum-binding kinetics, and deploying delivery strategies. Mechanistic studies in biologically relevant media (protein-rich buffers, intracellular uptake/ROS assays) and broader selectivity panels will be essential to deconvolute silver(I)-specific from ligand-driven effects and to advance this scaffold toward more selective antimicrobial candidates.

## Figures and Tables

**Table 1:** Thermodynamic parameters for the dissociation and substitution reactions of the silver(I) complex in water and DMSO.

Reaction	Δ*_r_G* (kcal mol^–1^)	*K*
{[Ag(NO_3_)(pqx-2ca)]_2_NO_3_}_(aq)_¯ ⇌ 2[Ag(NO_3_)(pqx-2ca)]_(aq)_ + NO_3(aq)_¯	−0.31	1.69
{[Ag(NO_3_)(pqx-2ca)]_2_NO_3_}_(DMSO)_¯ ⇌ 2[Ag(NO_3_)(pqx-2ca)]_(DMSO)_ + NO_3(DMSO)_¯	−0.22	1.44
[Ag(NO_3_)(pqx-2ca)]_(DMSO)_ + DMSO ⇌ [Ag(DMSO)(pqx-2ca)]^+^_(DMSO)_ + NO_3(DMSO)_¯	28.4	1.70 × 10^−21^

**Table 2:** Comparison of experimental and theoretical electronic transitions of the silver(I) complex with the pqx-2ca ligand.

Compound	Experimental λ (nm)	TD-B3LYP-D3BJ/6-311++ G (d,p)		
		λ (nm)	Oscillator Strength (f)	Major Transitions
[Ag(NO_3_)(pqx-2ca)]_n_	316	314	0.069	HOMO-1→LUMO+1 (70%)
	327	320	0.190	HOMO-6→LUMO (64%)

**Table 3:** Binding constants of the silver(I) complex and the pqx-2ca ligand with BSA.

Compound	*K_sv_* (M^−1^)	Hypochromism (%)	*K_q_* (M^−1^s^−1^)	*K_A_* (M^−1^)	*n*
[Ag(NO_3_)(pqx-2ca)]_n_	(4.63 ± 0.01) × 10^4^	43.1	4.63 × 10^12^	3.31 × 10^4^	0.97
pqx-2ca	(2.83 ± 0.05) × 10^4^	31.7	2.83 × 10^12^	3.24 × 10^4^	1.01

**Table 4:** Interaction of the [Ag(NO_3_)(pqx-2ca)] complex with BSA assessed by molecular docking simulations at three distinct binding sites. The table presents key thermodynamic parameters, including binding free energy (Δ*G*_bind_, kcal mol^−1^) and inhibition constants (*K*_i_, µM), as well as individual contributions from specific energy components: total internal energy (Δ*G*_total_), torsional energy (Δ*G*_tor_), unbound system energy (Δ*G*_unb_), electrostatic interactions (Δ*G*_elec_), the sum of dispersion and repulsion forces (Δ*G*_vdw_), hydrogen bonding (Δ*G*_hbond_), and desolvation energy (Δ*G*_desolv_).

Conformations	Δ*G_bind_*	*K*_i_ (µM)	Δ*G_inter_*	Δ*G_vdw+hbond+desolv_*	Δ*G_elec_*	Δ*G_total_*	Δ*G_tor_*	Δ*G_unb_*
[Ag(NO_3_)(pqx-2ca)]								
BSA-1	−8.23	0.92701	−10.43	−8.23	−2.19	−0.87	2.20	−0.87
BSA-2	−8.32	0.79149	−10.52	−9.70	−0.82	−0.69	2.20	−0.69
BSA-3	−11.74	0.00248	−13.94	−12.43	−1.50	−0.73	2.20	−0.73

**Table 5:** Binding constants of pqx-2ca and its silver(I) complex with the EthBr-ct-DNA and Hoe-ct-DNA systems.

Compound		*K_sv_* (M^−1^)	Hypochromism (%)	*K_q_* (M^−1^s^−1^)	*K_A_* (M^−1^)	*n*
EthBr	[Ag(NO_3_)(pqx-2ca)]_n_	(8.72 ± 0.01) × 10^2^	8.6	8.72 × 10^10^	1.97 × 10^2^	0.83
Hoe	pqx-2ca	(4.38 ± 0.02) × 10^3^	21.0	4.38 × 10^11^	1.89 × 10^8^	2.20
	[Ag(NO_3_)(pqx-2ca)]_n_	(9.82 ± 0.02) × 10^3^	34.0	9.82 × 10^11^	6.95 × 10^7^	2.00

**Table 6:** Interaction of the [Ag(NO_3_)(pqx-2ca)] complex with the hexanucleotide d(CGATCG)_2_ (PDB ID: 1Z3F) and canonical B-DNA (PDB ID: 1BNA) was assessed by molecular docking simulations. The table presents key thermodynamic parameters, including binding free energy (Δ*G*_bind_, kcal/mol) and inhibition constants (*K*_i_, µM), as well as individual contributions from specific energy components: total internal energy (Δ*G*_total_), torsional energy (Δ*G*_tor_), unbound system energy (Δ*G*_unb_), electrostatic interactions (Δ*G*_elec_), the sum of dispersion and repulsion forces (Δ*G*_vdw_), hydrogen bonding (Δ*G*_hbond_), and desolvation energy (Δ*G*_desolv_).

Complex	Δ*G_bind_*	*K*_i_ (µM)	Δ*G_inter_*	Δ*G_vdw+hbond+desolv_*	Δ*G_elec_*	Δ*G_total_*	Δ*G_tor_*	Δ*G_unb_*
**1Z3F**								
[Ag(NO_3_)(pqx-2ca)]	−7.29	4.54	−9.48	−9.42	−0.07	−0.48	2.20	−0.48
**B-DNA**								
[Ag(NO_3_)(pqx-2ca)]	−7.91	1.60	−10.10	−8.67	−1.43	−1.71	2.20	−1.71

**Table 7:** Antimycobacterial activity of the silver(I) complex and the pqx-2ca ligand expressed as minimum inhibitory concentrations (MIC, µg/mL (µM)) in comparison with that of the standard antibiotics.

	*M. tuberculosis H37Ra* *	M. smegmatis *	M. aurum *	M. avium *	M. kansasii *
pqx-2ca	15.625 (53.6)	≥500	≥500	>500	>500
[Ag(NO_3_)(pqx-2ca)]_n_	15.625 (33.9)	1.98 (4.3)	1.98 (4.3)	7.81 (16.9)	3.91 (8.5)
isoniazid	0.25	1.56	3.91	1000	25
rifampicin	0.0015625	12.5	0.39	0.125	0.025
ciprofloxacin	0.25	0.125	0.015625	1.56	0.25

* M. tuberculosis H37Ra (ATCC 9431); *M. smegmatis* (ATCC 607); *M. aurum* (ATCC 23366); *M. avium* (ATCC 25291); *M. kansasii* (ATCC 12478).

**Table 8:** Interaction of the [Ag(NO_3_)(pqx-2ca)] complex with the active sites of the enoyl-acyl carrier protein reductase (InhA) and Mycobacterial membrane protein Large 3 (MmpL3). The table presents key thermodynamic parameters, including binding free energy (Δ*G*_bind_, kcal mol^−1^) and inhibition constants (*K*_i_, µM), as well as individual contributions from specific energy components: total internal energy (Δ*G*_total_), torsional energy (Δ*G*_tor_), unbound system energy (Δ*G*_unb_), electrostatic interactions (Δ*G*_elec_), the sum of dispersion and repulsion forces (Δ*G*_vdw_), hydrogen bonding (Δ*G*_hbond_), and desolvation energy (Δ*G*_desolv_).

Compound	Δ*G_bind_*	*K*_i_ (µm)	Δ*G_inter_*	Δ*G_vdw+hbond+desolv_*	Δ*G_elec_*	Δ*G_total_*	Δ*G_tor_*	Δ*G_unb_*
**InhA**								
[Ag(NO_3_)(pqx-2ca)]	−7.95	1.48	−10.15	−10.23	0.08	−0.57	2.20	−0.57
INH1	−7.98	1.43	−9.90	−9.83	−0.07	−0.89	1.92	−0.89
**MmpL3**								
[Ag(NO_3_)(pqx-2ca)]	−9.10	0.21	−11.30	−11.53	0.23	−0.94	2.20	−0.94
INH2	−10.93	0.98	−13.40	−11.48	−1.91	−0.49	2.47	−0.49

## Data Availability

The experimental data used to support the findings of this study are available on request from the corresponding author. A sample of the silver(I) complex is available from the authors.

## Supplementary Materials

The following supporting information can be downloaded at: https://www.mdpi.com/article/10.3390/pharmaceutics18020169/s1. CCDC 2493068 contains the supplementary crystallographic data for this paper. These data can be obtained free of charge from The Cambridge Crystallographic Data Centre via www.ccdc.cam.ac.uk/data_request/cif. Figure S1. The stability of the silver(I) complex monitored by UV-Vis spectrophotometry at room temperature in DMSO over a 48 h period. Figure S2: Cyclic voltammograms of the silver(I) salt and its complex recorded at a GC electrode, in DMSO and 0.1 M TBAHP as the supporting electrolyte at a scan rate of 50 mVs^−1^. Figure S3: Fluorescence emission spectra of BSA in the absence and presence of increasing concentrations of the pqx-2ca ligand. The arrow indicates the changes in fluorescence intensity with increasing compound concentration. Inserted graph: Stern–Volmer plots of *F_0_*/*F* vs. [compound]. Figure S4: Fluorescence emission spectra of the EthBr-ct-DNA system in the absence and presence of increasing concentrations of the silver(I) complex. The arrow indicates the changes in fluorescence intensity with increasing complex concentration. Inserted graph: Stern–Volmer plots of *F_0_*/*F* vs. [compound]. Figure S5: Fluorescence emission spectra of the Hoe-ct-DNA system in the absence and presence of increasing concentrations of the pqx-2ca ligand. The arrow indicates the changes in fluorescence intensity with increasing compound concentration. Inserted graph: Stern–Volmer plots of *F_0_*/*F* vs. [compound]. Table S1: Calculated ^13^C and ^1^H NMR chemical shift values (ppm) for the [Ag(NO_3_)(pqx-2ca)] complex. Table S2: Details of the crystal structure determination for the [Ag(NO_3_)(pqx-2ca)]ₙ complex.
