# Supplementary material for: Polynuclear Silver(I)–Quinoxaline Complex: Comprehensive Structural Characterization, Antimycobacterial Properties and DNA/BSA Binding Study
Source: Pharmaceutics. 2026 Jan 27;18(2):169. doi: 10.3390/pharmaceutics18020169 (PMC12944352; doi:10.3390/pharmaceutics18020169)
Supplement: Supplementary file 1 [file pharmaceutics-18-00169-s001.zip › pharmaceutics-4098080-supplementary.pdf]

pharmaceutics

Supplementary Materials: Polynuclear Silver(I)–Quinoxaline Complex: Comprehensive Structural Characterization, Antimycobacterial Properties and DNA/BSA Binding Study

Ghada Bouz[1], Nevena Lj. Stevanović, Marta Počkaj, Tina P. Andrejević, Iztok Turel, Ondřej Janďourek, Klára Konečná, Žiko Milanović, Kristina Milisavljević, Biljana Đ. Glišić

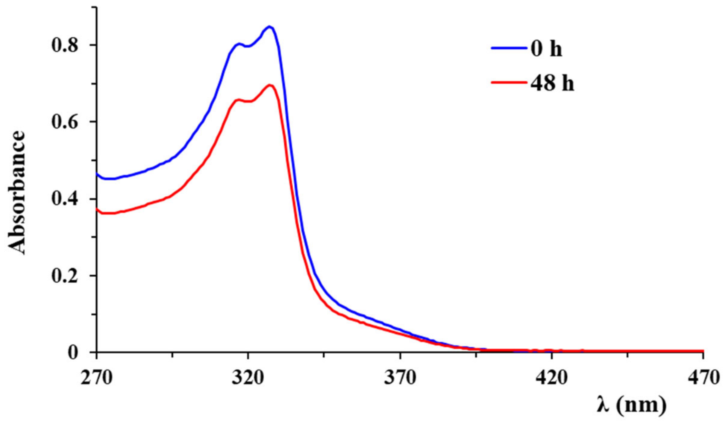

Figure S1. The stability of the silver(I) complex monitored by UV-Vis spectrophotometry at room temperature in DMSO over a 48 h period.

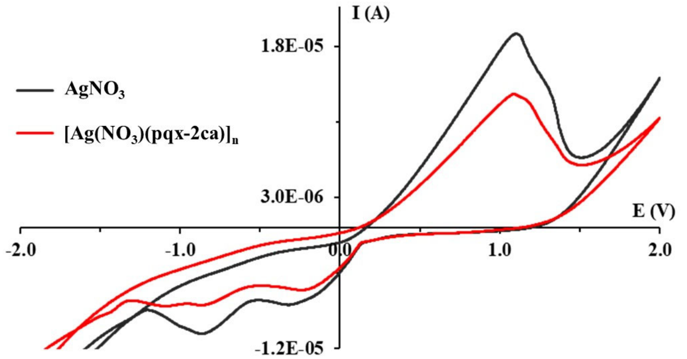

Figure S2. Cyclic voltammograms of the silver(I) salt and its complex recorded at a GC electrode, in DMSO and 0.1 M TBAHP as the supporting electrolyte at a scan rate of 50 mVs^{-1}.

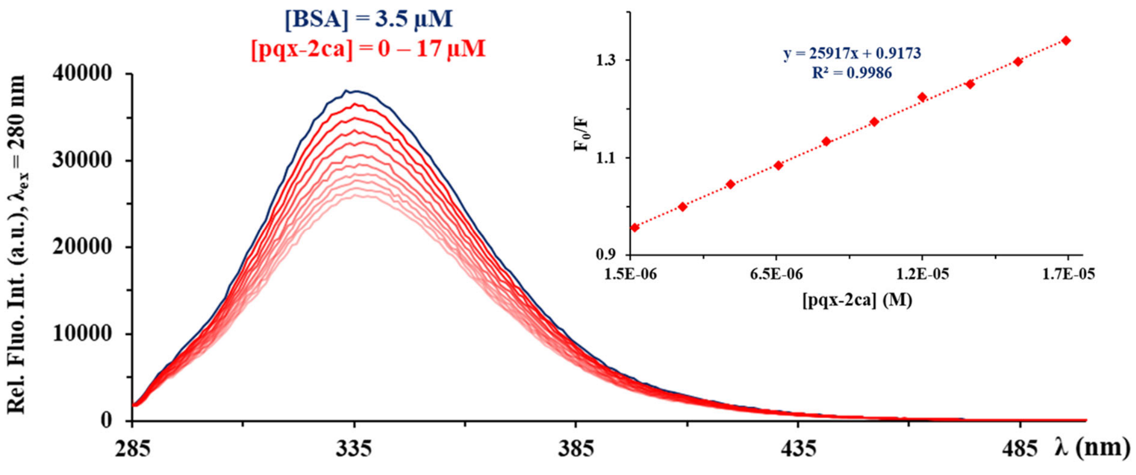

Figure S3. Fluorescence emission spectra of BSA in the absence and presence of increasing concentrations of the pqx-2ca ligand. The arrow indicates the changes in fluorescence intensity with increasing compound concentration. Inserted graph: Stern–Volmer plots of F_0/F vs. [compound].

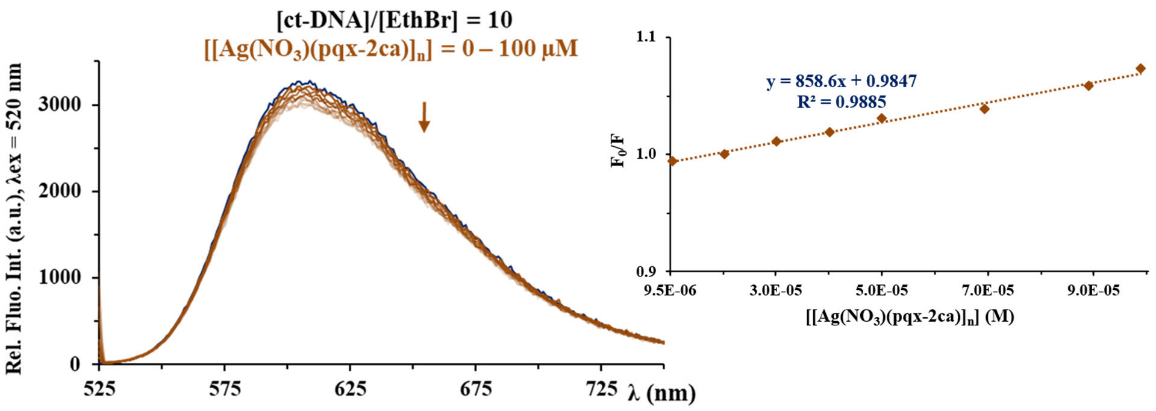

Figure S4. Fluorescence emission spectra of the EthBr-ct-DNA system in the absence and presence of increasing concentrations of the silver(I) complex. The arrow indicates the changes in fluorescence intensity with increasing complex concentration. Inserted graph: Stern–Volmer plots of F_0/F vs. [compound].

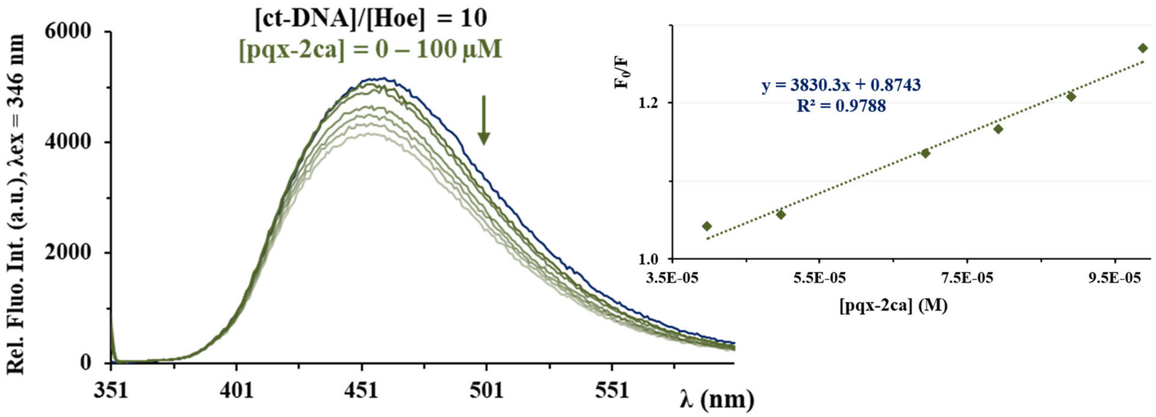

Figure S5. Fluorescence emission spectra of the Hoe-ct-DNA system in the absence and presence of increasing concentrations of the pqx-2ca ligand. The arrow indicates the changes in fluorescence intensity with increasing compound concentration. Inserted graph: Stern–Volmer plots of F_0/F vs. [compound].

Table S1. Calculated ^{13}C and ^{1}H NMR chemical shift values (ppm) for the [Ag(NO_3)(pqx-2ca)] complex.

Position	Theoretical values	
	^{13}C NMR (ppm)	^{1}H NMR (ppm)
C9	155.8	-
C3 (H3)	143.0	9.83
C2	142.2	-
C8a	139.6	-
C4'	139.1	-
C4a	137.3	-
C6 (H6)	131.6	8.51
C7 (H7)	129.3	8.51
C8 (H8)	127.7	8.64
C5 (H5)	126.3	8.71
C9'(H9')/C5'(H6')	124.4	7.68
C8'(H8')/C6'(H6')	124.1	7.71
C7'(H7')	121.6	7.59
C1'(H1')	42.8	3.85
C2'(H2')	38.9	2.84
C3'(H3')	37.3	1.98

Table S2. Details of the crystal structure determination for the $[Ag(NO_3)(pqx\text{-}2ca)]_n$ complex.

**Crystal data**	
Formula	$C_{18}H_{17}AgN_4O_4$
M_r	461.22
Cell setting, space group	Monoclinic, $P2_1/n$
a (Å)	17.9477(11)
b (Å)	5.3967(3)
c (Å)	19.2791(14)
β (°)	115.383(8)
V (Å^3)	1687.1(2)
Z	4
D_x (Mg m^{-3})	1.816
μ (mm^{-1})	1.230
F(000)	928
Crystal form, colour	Prism, colorless
Crystal size (mm^3)	0.05 × 0.10 × 0.25
**Data collection**	
T (K)	150(2)
No. of measured, independent and observed reflections	9371, 4435, 3337
R_{int}	0.0355
**Refinement**	
R (on F_{obs}), wR (on F_{obs}), S	0.0339, 0.0676, 1.024
No. of contributing reflections	4435
No. of parameters	248
No. of restraints	0
$\Delta\rho_{max}$, $\Delta\rho_{min}$ (eÅ^{-3})	0.509, −0.881

$R = \sum||F_o| - |F_c||/\sum|F_o|$; $wR_2 = \{\sum[w(F_o^2 - F_c^2)^2]/\sum[w(F_o^2)^2]\}^{1/2}$; $S = \{\sum[w(F_o^2 - F_c^2)^2]/(n - p)\}^{1/2}$ where n is the number of independent reflections and p is the total number of parameters refined.
