# Supplementary material for: Polynuclear Silver(I)–Quinoxaline Complex: Comprehensive Structural Characterization, Antimycobacterial Properties and DNA/BSA Binding Study
Source: Pharmaceutics. 2026 Jan 27;18(2):169. doi: 10.3390/pharmaceutics18020169 (PMC12944352; doi:10.3390/pharmaceutics18020169)

checkCIF/PLATON report

Structure factors have been supplied for datablock(s) shelx

THIS REPORT IS FOR GUIDANCE ONLY. IF USED AS PART OF A REVIEW PROCEDURE FOR PUBLICATION, IT SHOULD NOT REPLACE THE EXPERTISE OF AN EXPERIENCED CRYSTALLOGRAPHIC REFEREE.

No syntax errors found. CIF dictionary Interpreting this report

Datablock: shelx

Bond precision: C-C = 0.0040 A Wavelength=0.71073

Cell:	a=17.9477(11)	b=5.3967(3)	c=19.2791(14)
	alpha=90	beta=115.383(8)	gamma=90
Temperature:	150 K		

	Calculated	Reported
Volume	1687.1(2)	1687.1(2)
Space group	P 21/n	P 21/n
Hall group	-P 2yn	-P 2yn
Moiety formula	C18 H17 Ag N4 O4	C18 H17 Ag N4 O4
Sum formula	C18 H17 Ag N4 O4	C18 H17 Ag N4 O4
Mr	461.23	461.22
Dx,g cm-3	1.816	1.816
Z	4	4
Mu (mm-1)	1.230	1.230
F000	928.0	928.0
F000'	924.77	
h,k,lmax	25,7,27	23,7,27
Nref	5089	4435
Tmin,Tmax	0.863,0.940	0.735,1.000
Tmin'	0.735	

Correction method= # Reported T Limits: Tmin=0.735 Tmax=1.000
AbsCorr = MULTI-SCAN

Data completeness= 0.871 Theta(max)= 30.342

R(reflections)= 0.0339(3337) wR2(reflections)= 0.0754(4435)

S = 1.024 Npar= 248

The following ALERTS were generated. Each ALERT has the format
test-name_ALERT_alert-type_alert-level.
Click on the hyperlinks for more details of the test.

Alert level G

```
PLAT004_ALERT_5_G Polymeric Structure Found with Maximum Dimension          1 Info
PLAT232_ALERT_2_G Hirshfeld Test Diff (M-X)  Ag1      --O23      .       11.2 s.u.
PLAT232_ALERT_2_G Hirshfeld Test Diff (M-X)  Ag1      --O26_b    .       23.7 s.u.
PLAT899_ALERT_4_G SHELXL2018 is Outdated   and Succeeded by SHELXL     2019/3 Note
PLAT910_ALERT_3_G Missing FCF Reflection(s) Below Theta(Min)[Deg]=       2.34 Note
               -1  0  1,   1  0  1,
PLAT912_ALERT_4_G Missing # of FCF Reflections Above STh/L=  0.600        605 Note
PLAT941_ALERT_3_G Average HKL Measurement Multiplicity ...........        2.1 Low
PLAT950_ALERT_5_G Calculated (ThMax) and CIF-Reported Hmax Differ           2 Units
PLAT956_ALERT_1_G Calculated (ThMax) and Actual (FCF) Hmax Differ           2 Units
PLAT969_ALERT_5_G The 'Henn et al.' R-Factor-gap value ...........      1.498 Note
              Predicted wR2: Based on SigI**2  5.04 or SHELX Weight  7.37
PLAT978_ALERT_2_G Number C-C Bonds with Positive Residual Density.          4 Info
```

---

0 **ALERT level A** = Most likely a serious problem - resolve or explain
0 **ALERT level B** = A potentially serious problem, consider carefully
0 **ALERT level C** = Check. Ensure it is not caused by an omission or oversight
11 **ALERT level G** = General information/check it is not something unexpected

1 ALERT type 1 CIF construction/syntax error, inconsistent or missing data
3 ALERT type 2 Indicator that the structure model may be wrong or deficient
2 ALERT type 3 Indicator that the structure quality may be low
2 ALERT type 4 Improvement, methodology, query or suggestion
3 ALERT type 5 Informative message, check

It is advisable to attempt to resolve as many as possible of the alerts in all categories. Often the minor alerts point to easily fixed oversights, errors and omissions in your CIF or refinement strategy, so attention to these fine details can be worthwhile. It is up to the individual to critically assess their own results and, if necessary, seek expert advice.

PLATON version of 26/09/2025; check.def file version of 20/09/2025

duplicate check

No duplication found

Datablock shelx - ellipsoid plot

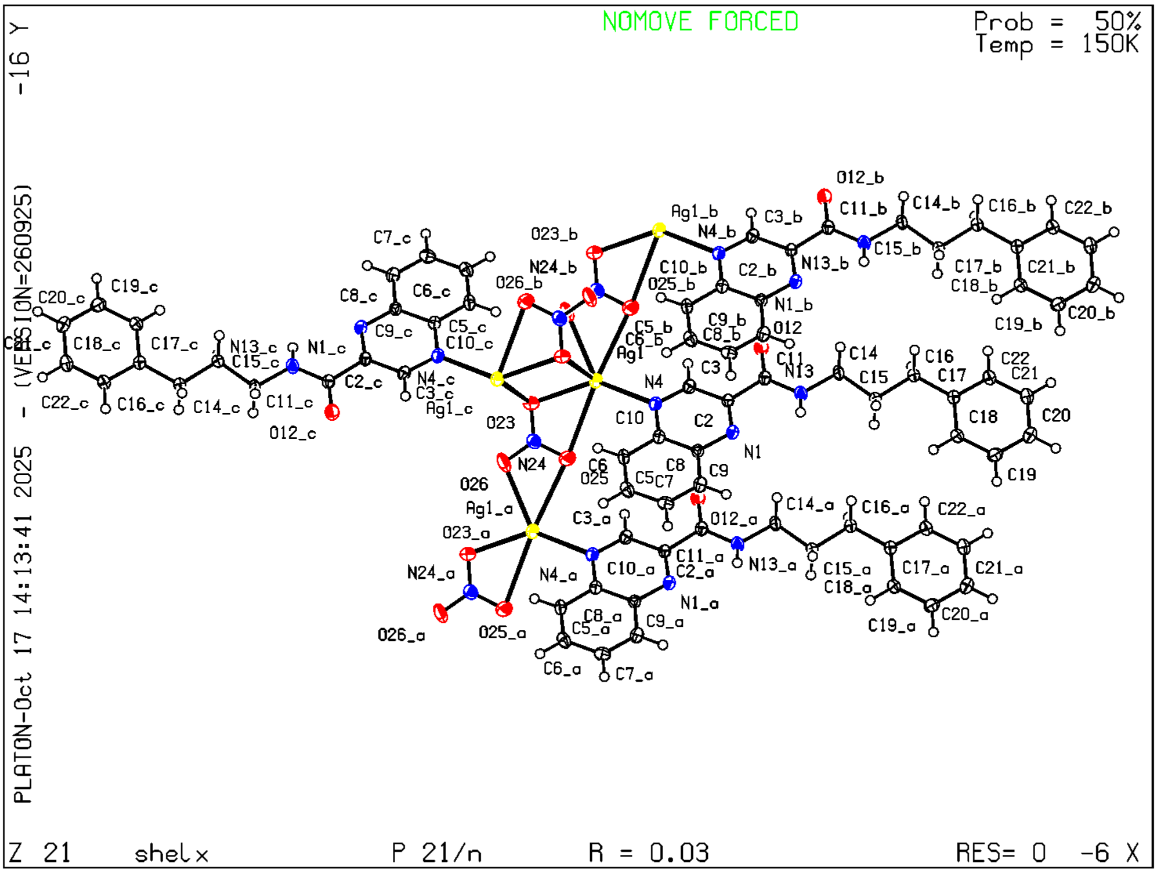

Supplement: Supplementary file 1 [file pharmaceutics-18-00169-s001.zip › checkcif_moc623.pdf]
